# Effectiveness of Mechanical Chest Compression Devices over Manual Cardiopulmonary Resuscitation: A Systematic Review with Meta-analysis and Trial Sequential Analysis

**DOI:** 10.5811/westjem.2021.3.50932

**Published:** 2021-07-19

**Authors:** Mack Sheraton, John Columbus, Salim Surani, Ravinder Chopra, Rahul Kashyap

**Affiliations:** * Trinity West Medical Center, Department of Emergency Medicine, Steubenville, Ohio; † Texas A&M University, Health Sciences Center, Corpus Christi, Texas; ‡ Mayo Clinic, Department of Anesthesiology and Critical Care, Rochester, Minnesota

## Abstract

**Introduction:**

Our goal was to systematically review contemporary literature comparing the relative effectiveness of two mechanical compression devices (LUCAS and AutoPulse) to manual compression for achieving return of spontaneous circulation (ROSC) in patients undergoing cardiopulmonary resuscitation (CPR) after an out-of-hospital cardiac arrest (OHCA).

**Methods:**

We searched medical databases systematically for randomized controlled trials (RCT) and observational studies published between January 1, 2000–October 1, 2020 that compared mechanical chest compression (using any device) with manual chest compression following OHCA. We only included studies in the English language that reported ROSC outcomes in adult patients in non-trauma settings to conduct random-effects metanalysis and trial sequence analysis (TSA). Multivariate meta-regression was performed using preselected covariates to account for heterogeneity. We assessed for risk of biases in randomization, allocation sequence concealment, blinding, incomplete outcome data, and selective outcome reporting.

**Results:**

A total of 15 studies (n = 18474), including six RCTs, two cluster RCTs, five retrospective case-control, and two phased prospective cohort studies, were pooled for analysis. The pooled estimates’ summary effect did not indicate a significant difference (Mantel-Haenszel odds ratio = 1.16, 95% confidence interval, 0.97 to 1.39, P = 0.11, I2 = 0.83) between mechanical and manual compressions during CPR for ROSC. The TSA showed firm evidence supporting the lack of improvement in ROSC using mechanical compression devices. The Z-curves successfully crossed the TSA futility boundary for ROSC, indicating sufficient evidence to draw firm conclusions regarding these outcomes. Multivariate meta-regression demonstrated that 100% of the between-study variation could be explained by differences in average age, the proportion of females, cardiac arrests with shockable rhythms, witnessed cardiac arrest, bystander CPR, and the average time for emergency medical services (EMS) arrival in the study samples, with the latter three attaining statistical significance.

**Conclusion:**

Mechanical compression devices for resuscitation in cardiac arrests are not associated with improved rates of ROSC. Their use may be more beneficial in non-ideal situations such as lack of bystander CPR, unwitnessed arrest, and delayed EMS response times. Studies done to date have enough power to render further studies on this comparison futile.

## INTRODUCTION

Sudden out-of-hospital cardiac arrests (OHCA) are significant causes of morbidity and mortality both in the US and worldwide. About 326,200 OHCAs are resuscitated annually by emergency medical services (EMS) with a survival rate of approximately 12% in the US.^1^ Early and high-quality cardiopulmonary resuscitation (CPR) has been identified as a critical factor for survival during resuscitation.^2^ To achieve high quality, the American Heart Association recommends a chest compression rate of 100–120 per minute and a compression depth of at least 5 centimeters during CPR.^1^ However, various challenges in the field settings threaten to make the CPR delivered by EMS personnel suboptimal. These include a lack of enough human resources, fatigue, competing tasks on arrival, and the challenge of continuing CPR in a moving ambulance.

In the early 2000s, two mechanical compression devices (AutoPulse [Zoll Medical Corporation, Chelmsford, MA] and LUCAS [Physio-Control/Jolife AB, Lund, Sweden]) were approved by the US Food and Drug Administration (FDA) to help surmount these challenges. The AutoPulse device is a load-distributing band device in which a wide band fits circumferentially around the chest wall. This band is automated to shorten and lengthen alternately to provide compressions. The LUCAS device belongs to a different category of piston devices: A piston mounted on a circumferential frame uses a power source to move up and down forcefully, simulating manual compressions.

Theoretically, these mechanical devices should help eliminate the problems associated with fatigue, manpower, and CPR consistency, whether in the field or during transport. They also help free up the ambulance crew for other tasks related to resuscitation. Studies done on porcine models have shown improved coronary perfusion and end-tidal CO_2_ achieved with mechanical compressions during transport.^3^ However, results from clinical trials have been conflicting. Some studies have shown a benefit, while others demonstrated no difference in outcomes using mechanical compressions. Our goal in this systematic review was to synthesize studies comparing outcomes from mechanical and manual CPR during OHCA regardless of presenting rhythm.

## METHODS

### Search Strategy

We used a prespecified protocol and a clear, reproducible plan for a literature search and synthesis as per the Preferred Reporting Items for Systematic Reviews and Metaanalyses (PRISMA) statement.^4^ The review protocol has not been registered. PubMed, Embase, Scopus, Google Scholar, CINAHL, and Cochrane databases were systematically searched for related articles published between January 1–October 1, 2020. In all electronic databases, the following search strategy was implemented, and these phrases were queried (in the title/abstract, keywords and their MeSH subheadings) with appropriate restrictions: “Cardiopulmonary resuscitation” AND “out-of-hospital cardiac arrests” AND “mechanical compression devices” AND “return of spontaneous circulation.” We scanned the included studies’ reference lists or relevant reviews identified through the search along with available gray literature to ensure saturation. We concluded the inquiry on November 5, 2020.

### Eligibility Criteria

We included peer-reviewed human studies of adult (age >18 years) cardiac arrests (CA) comparing mechanical vs manual compression outcomes in out-of-hospital settings, reported in English, only from North American and European countries with comparable advanced EMS. We excluded case reports, narrative reviews, commentaries, letters, abstracts, mannequin/animal studies, and studies using mechanical devices other than Autopulse or LUCAS for resuscitation. The cases were patients in non-trauma settings who received chest compressions with a mechanical device, and controls included similar patients who received them manually. Authors (MS, JC) participated in each phase of the review (screening, eligibility, and inclusion). Titles and abstracts were individually evaluated by two authors (MS, JC) to identify and assess key articles. Two authors (MS, JC) independently reviewed the entire manuscript and registered justification for exclusions. Discrepancies were addressed by arbitration by a third reviewer.

### Outcome

We chose ROSC for 20 minutes or more after resuscitation in an OHCA as the primary outcome. Our presumption was this outcome most directly reflects the acute effects of the CPR. Long-term outcomes, such as survival to discharge and neurological outcomes, were more likely influenced by post-resuscitation care.

### Data Collection

Two authors (MS, JC), using a standardized data extraction method, extracted information from each study independently; conflicts were resolved by consensus. The following data points were extracted: name of the first author; year of study; sample size; number of participants per treatment arm; study design; type of device used; time delay in mechanical compressions; inclusion and exclusion criteria; average age, gender (percentage female); percentage of witnessed arrests; the percentage receiving bystander CPR; percentage with an initial shockable rhythm; time in minutes of EMS arrival; and primary outcome (ROSC).

### Risk of Bias Assessment

We used the Newcastle-Ottawa scale (NOS) to measure the risk of bias in observational studies.^5^ The following classes were rated per study: low bias risk (8–9 points); moderate bias risk (5–7 points); and high bias risk (0–4 points). The modified Cochrane risk of bias tool was used to assess risk of bias in randomized controlled trials (RCT).^6^ This tool considers selection, performance, detection, attrition, reporting, and other biases. Three reviewers (MC, JC, RC) evaluated the likelihood of bias independently, and any conflict was resolved by consensus.

### Statistical Analysis

The RCTs and observational studies included compared outcomes of mechanical and manual compressions during OHCA resuscitations. Meta-analysis was performed for studies reporting ROSC of patients in both groups assuming independence of results from other reported endpoints. Due to anticipated heterogeneity, we calculated summary statistics using a random-effects model. In all cases, meta-analyses were performed using the Mantel–Haenszel (M-H) method for dichotomous data to estimate pooled odds ratios (OR). Statistical heterogeneity was assessed using Q-values and I^2^ statistics. We performed the metanalysis, metaregression, and assessment of publication bias using Comprehensive Meta-Analysis software (Biostat Inc., Englewood, NJ).^7^

Next, we performed trial sequence analysis (TSA) to assess the quality of available data and conclusions from the meta-analysis. This applies sequential monitoring boundaries to a meta-analysis by calculating sample sizes contributed by included studies, known as information size (IS). A Z-curve is constructed by cumulative evidence of trials added over time. If this curve crosses the alpha boundary of significance, then sufficient evidence favoring the intervention has been achieved. However, if it crosses the futility boundary, the cumulative evidence is adequate to indicate no effect for the intervention examined.^8^ Applying TSA boundaries guard against the risk of false-positive (type-I error) and false-negative (type-II error) results. We maintained the two-sided type-I error rate at 5% (alpha boundary) and calculated the required IS with 80% power, assuming a 20% relative risk reduction for mechanical compressions. We conducted the analysis using TSA software, Copenhagen Trial Unit, version 0.9.5.10 Beta^9^ (Centre for Clinical Intervention Research, Copenhagen, Denmark).

To explore intrinsic differences between studies expected to influence the effect size, we performed random effects (maximum likelihood method) univariate and multivariate meta-regression analyses. The potential sources of variability defined a priori were average age, gender (percentage female), percentage of witnessed arrests, the percentage receiving bystander CPR, percentage with an initial shockable rhythm, and time in minutes of EMS arrival.

## RESULTS

### Study Selection

**Figure 1:**
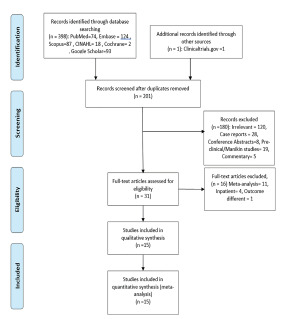
Preferred Reporting Items for Systematic Reviews and Meta-Analyses (PRISMA) flow diagram.

The search identified 398 articles (Figure 1), which were culled to 201 potentially eligible studies after removing duplicates. No articles were added from a manual search of references; however, one was added through gray literature sources (www.clinicaltrials.gov). In all, we excluded 180 studies (120 irrelevant to the present context, 28 case reports, eight conference abstracts without full publication, 19 pre-clinical or mannequin studies, and five commentaries) after review of their titles and abstracts. We excluded 16 after full-text assessments of the remaining 31 articles because 11 were meta-analyses, four were studies conducted in inpatient settings, and one used cerebral perfusion as the resuscitation outcome.^10^ And we excluded all four articles reporting inpatient CA including one by Koster et al that considered resuscitations in the emergency department as OHCA resuscitations.^11^ A resulting total of 15 studies were included in meta-analysis for the primary outcome. Altogether, these studies consisted of 8685 resuscitations in the mechanical compression arm and 9789 in the manual compression arm.

### Study Characteristics

Of the 15 studies selected (Table 1), six were RCTs and nine were observational studies. Of the RCTs, three were conducted using the AutoPulse device (n = 5119),^12^ ^13^ ^14^ and the remainder involved the LUCAS device (n = 7209).^15^ ^16^ ^17^ Of the nine observational studies, two were cluster RCTs,^18^ ^19^ two were phased prospective cohort studies,^20^ ^21^ and the rest were retrospective case-control studies.^22^ ^23^ ^24^ ^25^ ^26^ Four of the observational studies used the LUCAS device (n = 3018), and the remaining five (n = 3508) used AutoPulse. All studies were published between 2006–2016 and had sample sizes ranging from 133 to 4471.

### Risk of Bias Assessment

Most observational studies were found to have a low or moderate risk of bias according to the NOS scale (Appendix 1). The studies that had a comparably higher risk of bias in the group failed to control for any confounding factors by design, thereby losing out on the “comparability” score.^26^ ^24^ The RCTs, however, collectively had a higher risk of bias (Appendix 2). Randomized sequence generation was adequately performed in only two trials,^13^ ^27^ and allocation sequence concealment was adequate in only one.^13^ Given the nature of the intervention, blinding participants in the field is not possible, resulting in increased performance bias in all studies. However, the PARAMEDIC trial achieved low assessor bias by blinding research nurses.^15^ Attrition bias was low overall for the ROSC outcome. Also noteworthy is that the ASPIRE trial had high “other” biases because it was stopped early due to interim evidence of worse outcomes in the intervention arm.^12^

### Primary Outcome

**Figure 2:**
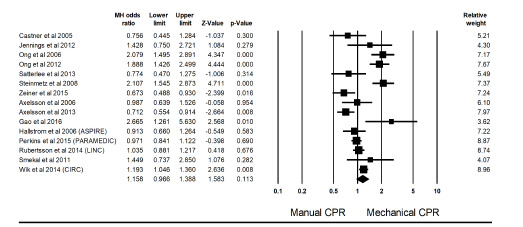
Forest plot for meta-analysis comparing manual vs mechanical cardiopulmonary resuscitation. Heterogeneity: Tau^2^=9.1%, SE= 0.056, I^2^=83.079%, df=14 (p=0.00), Q=82.74 *ROSC*, return of spontaneous circulation.

Meta-analysis summary statistics showed that mechanical chest compressions did not significantly improve ROSC (relative risk (RR) 0.80, 95% confidence interval (CI), 0.61, 1.04, *P* = 0.10; I^2^ = 65%) (Figure 2) when compared with manual chest compressions in patients undergoing resuscitation after OHCA (M–H odds ratio (OR) = 1.16, 95% CI, 0.97, 1.39, *P* = 0.11). Heterogeneity was high with I^2^ = 83.07% and Q-value of 82.74.

### Multivariate Meta-regression Model

**Figure 3:**
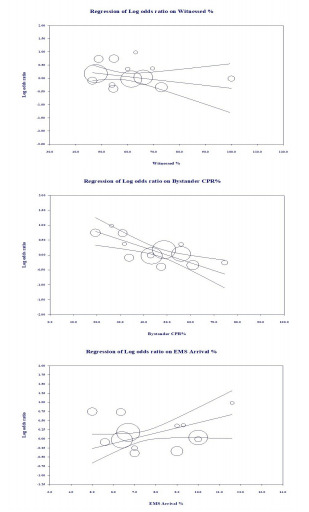
Results of meta-regression. Y = 1.0934 − 0.0121 * Witnessed % − 0.0239 * Bystander CPR% + 0.1079 * EMS Arrival %

Multivariate meta-regression, performed to try to explain high between-studies variations in association between ROSC and mechanical vs manual CPR, revealed that the following covariates had an effect: average age (log OR = −0.02, standard error [SE] = 0.02); gender distribution (log OR = 0.02, SE = 0.01); percentage of witnessed arrests (log OR = 0.01, SE= 0.01); percentage of bystander CPR (log OR = −0.03, SE = 0.00); time lag for EMS arrival (log OR = 0.14, SE = 0.04); and percentage of shockable rhythm (log OR = −0.02, SE = 0.01) (Appendix 3). Only three of these, the percentage of witnessed OHCA, the percentage receiving bystander CPR, and time lag for EMS arrival, achieved significance at the *P* <0.05 level (Figure 3). There was a decreasing benefit of mechanical over manual CPR for ROSC with increasing percentages of witnessed arrests and increasing percentage of bystander CPR; however, the benefit increased with delays in EMS response. Altogether, they explained 100% of the between-study heterogeneity.

### Trial Sequence Analysis

**Figure 4:**
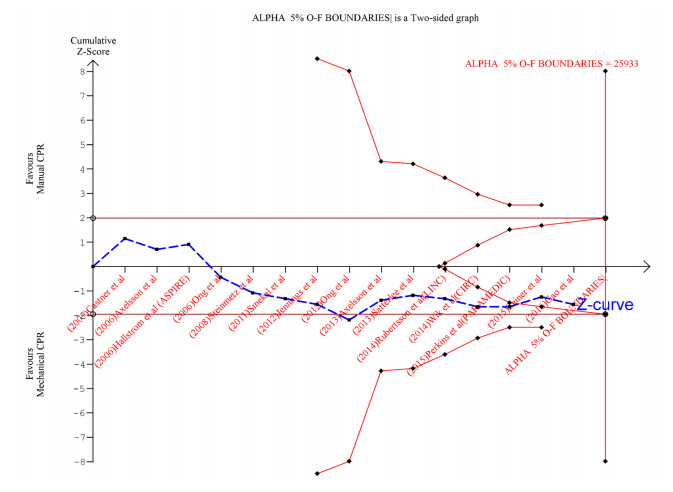
Trial sequential analysis for favorable return of spontaneous circulation (ROSC) outcome. The diversity-adjusted information size (sample size) equal to 25,933 (vertical red line). The cumulative Z-curve (blue line with small black squares representing each trial) failed to cross both the traditional (horizontal maroon line), trial sequential monitoring boundary (concave red line). But it crosses the futility boundary (red triangle), indicating firm evidence supporting the lack of favorable ROSC outcomes with mechanical compressions during cardiopulmonary resuscitation.

Applying the TSA boundaries to favorable ROSC outcomes showed that an IS of 25933 was required to achieve 80% power. This IS could not be acquired from the pooled studies. However, the Z-curve crossed the futility boundary even though it failed to cross the conventional or TSA boundaries (Figure 4). This indicates firm cumulative power from the available literature to support the lack of association between outcome and intervention.

### Publication Bias

**Figure 5:**
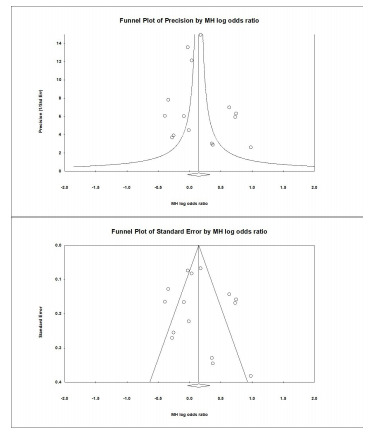
Funnel plots of precision and standard error demonstrating publication bias.

Visual inspection of the SE and precision plots for the analysis (Figure 5) suggest asymmetry with an under-representation of negative studies with lesser precision and smaller effect sizes. Classic fail-safe N analysis (alpha = 0.05) placed the number of missing studies at 31. Corroborating inspection findings, Egger’s regression test with the null hypothesis of no small study effects was not rejected at *P* <0.05 (estimated bias coefficient = 0.75 ± 1.31). Overall, we assessed some risk of publication bias, especially for smaller studies.

## DISCUSSION

### Summary of Findings

The overall proportion of successful resuscitations in OHCA remains dismal. Although mechanical chest compression devices have been around for the last 40 years, we have seen a recent surge in interest because of FDA and American Hospital Association approvals of their usage, resuscitation guidelines stressing correct delivery of compressions, and lighter and more portable devices, making them more user-friendly and less time-consuming.^28^

We synthesized studies performed in the last 20 years only, assuming that the effects of resuscitations can be captured only in the context of guiding protocols. Because earlier protocols for chest compressions were different from present-day protocols, this would have introduced pipeline bias into the analysis. To maintain uniformity of guidelines, we excluded studies from outside of North America and Europe. Finally, we excluded studies using non-FDA approved compression devices such as the Thumper (Michigan Instruments, Grand Rapids, MI) and mechanical life-vest or nonautomated ones such as the impedance threshold device, assuming non-comparability of outcomes.^29^

Our analysis failed to demonstrate a significant advantage in using mechanical devices. Meta-regression, however, showed greater benefit over manual CPR in the absence of witnesses at the time of arrest, lack of bystander CPR, or delay in EMS arrival. Thus, we derive that they do have a place in non-ideal resuscitations where early initiation of quality CPR has not been possible. Trial sequence analysis suggested that sufficient evidence has already been accumulated to refute superiority of mechanical over manual compressions convincingly. It indicates the futility in further investigations into the improvement of ROSC with use of mechanical devices.

### Strengths

The main strength of our analysis was the inclusion of a large number of studies that increased its statistical power. To our knowledge, this is the first meta-analysis to use TSA to confirm the sufficiency of accumulated evidence. Moreover, the study has high external validity from including studies from various European and North American countries.

### Prior Studies

Most notable, of the prior studies synthesizing evidence for or against mechanical compression devices, is the Cochrane systematic review by Wang et al published in 2011 and updated twice in 2016 and 2018.^30^ They included all RCTs that were ever done and concluded that CPR with mechanical devices did not result in improved ROSC. Ong et al synthesized 10 studies that included only one RCT, with four evaluating compression adequacy and six evaluating survival outcomes. They reported that, although compressions adequacy was better, outcomes were similar to those of mechanical devices.^31^ Westfall et al synthesized 11 observational and one pilot RCT and concluded that mechanical devices with load distributing bands had better ROSC outcomes than manual CPR.^32^ Although they also performed meta-regression, unlike our study, none of their covariates significantly accounted for between-study variations. However, this study was funded by Zoll, the manufacturers of the AutoPulse device, and therefore its findings may be called into question. Gates et al included five RCTs in their study and performed subgroup analysis for the type of device used, either LUCAS or AutoPulse. They found no difference in outcomes for either device vs manual compressions.^33^

Bonnes et al performed a subgroup analysis of five RCTs and 15 observational studies. While observational studies showed some advantages from mechanical compressions, the RCTs did not indicate any difference. Contrary to our meta-regression analysis, they showed a decreasing benefit of mechanical compressions with longer EMS response delay.^34^ Tang et al analyzed the same fivc RCTs but conducted subgroup analysis for outcomes. They found worse outcomes for ROSC from using mechanical CPR and advised against it.^35^ Li et al summarized the effects from nine studies, both RCTs and observational in the out-patient settings, and three in the inpatient settings. They concluded that manual compressions were more likely to achieve ROSC when compared to load-distributing bands.^36^ Khan et al performed a Bayesian network meta-analysis of seven RCTs comparing the safety and efficacy of both types of CPR; manual compression was found to be more effective than AutoPulse and had a lesser risk of adverse effects.^37^ Zhu et al synthesized nine RCTs and six cohort studies and found no difference in a variety of resuscitation outcomes between manual and mechanical CPR. Their subgroup analyses for the type of device used (AutoPulse and LUCAS) also resulted in a similar conclusion as ours.^38^

Of note, this metanalysis attains the largest sample size to date, primarily from including the Buckler et al observational study that uses the CARES registry.^39^ Lameijer et al and Couper et al conducted meta-analyses of studies conducted only in inpatient settings and reported a definite advantage using mechanical compression.^40^,^41^ Appendix 4 contains studies that we considered but eventually excluded from our meta-analysis with the respective exclusion criteria.

## LIMITATIONS

The primary limitation was the necessity for using observational studies. Even though these are considered inferior to RCTs, the nature of our question makes both types comparable. Of all the RCTs we considered, none included blinding of the participant because it is impossible for EMS personnel not to know when they are using a compression device and when they are performing manual compressions. Selection bias in RCTs was comparable to observational studies, with most using clusters rather than true randomizations. Because ROSC was considered as the only outcome, there was almost no attrition bias in either RCTs or observational studies. One could argue about inherent calculation bias in observational studies; however, almost all considered here were of high quality with low risk of bias.

Second, the design of each study was different. There was no uniform protocol for CPR administration and an absence of data regarding CPR quality in all studies. Indicators of quality CPR such as cerebral perfusion, resuscitative tracheoesophageal echocardiography, and end tidal CO_2_ were absent from all studies considered. Third, there were also differences in composition of teams of first responders. The initial response could occur with teams of only police/firefighters or with more advanced paramedics/physician teams. There were differences in regional policies governing such teams and training regarding usage of mechanical compression devices.

Fourth, field conditions for each study were different. Logistic differences such as location of the arrest, distance from the hospital, traffic negotiated by ambulance, and safety of manual compressions in transit were not accounted for. Fifth, equipment factors for the ambulance and compression devices were very different. The type of ambulance, its average capacity and speed, time to deployment of mechanical compression after starting resuscitation, the possibility of a patient randomized to the mechanical arm not fitting in the device could have added to the heterogeneity. Finally, even though it made sense to include only nonpregnant adults because of the uniform size of the machines, stratification by age and gender would have yielded more credible results.

## CONCLUSION

The use of mechanical devices during CPR does not lead to improvement in ROSC outcomes even though it improves the quality of CPR. Nevertheless, we do recommend the availability of these devices as an option to EMS personnel as insurance against fatigue, lack of manpower, and other situations in the field precluding early initiation of high-quality manual CPR. We established futility of additional trials to determine the utility in the context of out-of-hospital cardiac arrest; nevertheless, we recommend high-quality random controlled trials in inpatient settings. Positive results from a recent pilot study for the COMPRESS trial by Couper et al has been an encouraging development in this direction.^42^

## Supplementary Information



## Figures and Tables

**Table 1:** Types of studies included.

Name	n	Device	Study	Exclusion criteria
Hallstrom et al 2006 (ASPIRE)	767	Autopulse	RCT	<18, Trauma, recent surgery, prisoners, DNR
Smekal et al 2011	149	LUCAS	RCT	<18, Trauma, pregnancy
Wik et al 2014 (CIRC)	4219	Autopulse	RCT	<18, Trauma, pregnancy, prisoner, DNR, large for device, EMS arrival >16 mins
Rubertsson et al 2014 (LINC)	2589	LUCAS	RCT	<18, Trauma, pregnancy
Perkins et al 2015 (PARAMEDIC)	4471	LUCAS	RCT	<18, Trauma, pregnancy
Gao et al 2016	133	Autopulse	Prospective RCT	<14>90, Trauma, pregnant, advanced cancer
Castner et al 2005	262	Autopulse	Retrospective case-control	None
Axelsson et al 2006	328	LUCAS	Cluster RCT	Witnessed OHCA, <18, trauma, pregnancy, hypothermia, intoxication, discharge, hanging, drowning, ROSC before arrival
Ong et al 2006	783	Autopulse	Phased prospective cohort	Trauma, <18, mentally disabled, prisoners, pregnant women
Steinmetz et al 2008	791	Autopulse	Retrospective case-control	None
Jennings et al 2012	286	Autopulse	Retrospective case-control	None
Ong et al 2012	1101	Autopulse	Phased prospective cohort	Trauma, <18, Non-cardiac
Satterlee et al 2013	572	LUCAS	Restrospective case series	Pregnant, <18, Non-cardiac
Axelsson et al 2013	1170	LUCAS	Cluster RCT	None
Zeiner et al 2015	948	LUCAS/Autopulse	Restrospective case-control	None

*RTC*, randomized controlled trial; *DNR*, do not resuscitate; *EMS*, emergency medical services; *OHCA*, out-of-hospital cardiac arrest.
