# Supplementary material for: Effectiveness of Mechanical Chest Compression Devices over Manual Cardiopulmonary Resuscitation: A Systematic Review with Meta-analysis and Trial Sequential Analysis
Source: West J Emerg Med. 2021 Jul 19;22(4):810–9. doi: 10.5811/westjem.2021.3.50932 (PMC8328162; doi:10.5811/westjem.2021.3.50932)
Supplement: Supplementary file 1 [file wjem-22-810-s001.docx]

**Appendix 1:** RoB 2 for risk of bias of RCTs

| Study | Allocation: generation  (Selection) | Allocation: concealment  (Selection) | Blinding: participants  (Performance) | Blinding: assessors  (Detection) | Outcome: complete (Attrition) | Outcome: selective  (Reporting) | Other bias |
| --- | --- | --- | --- | --- | --- | --- | --- |
| Hallstrom et al 2006 (ASPIRE) | Unclear | Unclear | High | Unclear | Low | Unclear | High |
| Smekal et al 2011 | Unclear | Unclear | High | Unclear | Low | Unclear | High |
| Wik et al 2014 (CIRC) | Unclear | Unclear | High | High | Low | Unclear | Unclear |
| Rubertsson et al 2014 (LINC) | Unclear | Low | High | Unclear | Low | Unclear | Unclear |
| Perkins et al 2015 (PARAMEDIC) | Low | Unclear | High | Low | Low | Unclear | High |
| Gao et al 2016 | Low | High | High | Unclear | Low | Unclear | Low |

**Appendix 2:** The NOS tool for risk of bias in observational studies

| Study | Selection | Comparability | Outcome | Score |
| --- | --- | --- | --- | --- |
| Castner 2005 | 4 | 2 | 3 | 9/9 |
| Axelsson 2006 | 3 | 1 | 3 | 7/9 |
| Ong 2006 | 4 | 2 | 3 | 9/9 |
| Steinmetz 2008 | 3 | 0 | 3 | 6/9 |
| Ong 2012 | 4 | 2 | 3 | 9/9 |
| Jennings 2012 | 3 | 2 | 3 | 8/9 |
| Axelsson 2013 | 4 | 2 | 3 | 9/9 |
| Satterlee 2013 | 4 | 0 | 3 | 7/9 |
| Zeiner 2015 | 3 | 1 | 3 | 7/9 |

**Appendix 3:** Main results for Random effects Maximum Likelihood metaregression covariates (Log Odds ratio and Z-distribution)

**
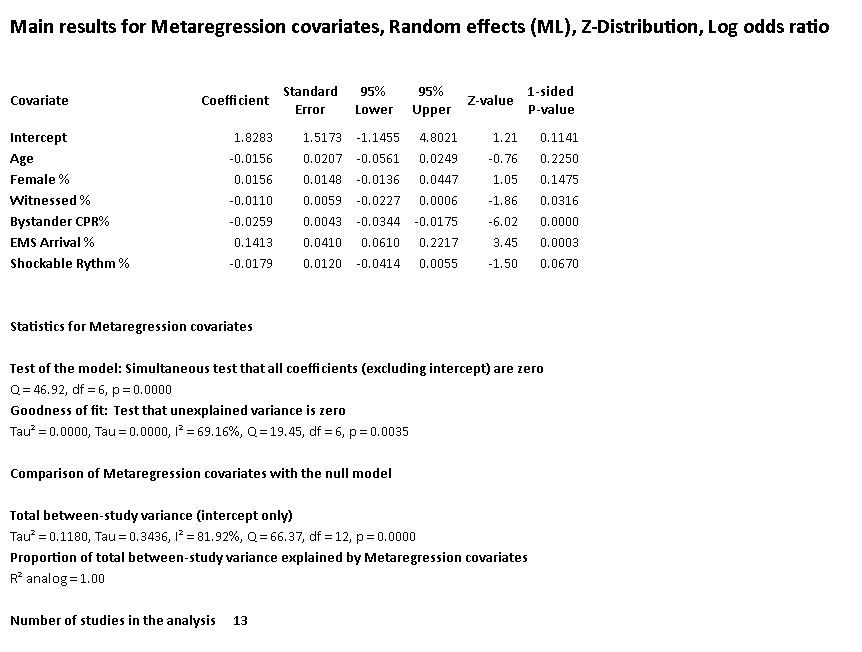
**

**Appendix 4**: Excluded studies with reasons for exclusion:

| **Name** | **n** | **Reason for exclusion** |
| --- | --- | --- |
| Box et al. 2008 | 87 | ACD- CPR* usage |
| Lairet et al. 2005 | 479 | Only abstract published |
| Plaisance et al. 1999 | 750 | ACD- CPR usage, publication period exclusion** |
| Skogvoll et al. 1999 | 431 | ACD- CPR usage, publication period exclusion |
| Wolcke et al. 2003 | 210 | ACD- CPR with ITD |
| Dickinson et al. 1999 | 20 | Thumper usage, publication period exclusion |
| Halperin et al. 1993 | 63 | Thoracic vest usage, publication period exclusion |
| Koster et al. 2017 | 374 | Safety of usage was the studied outcome |
| Lu et al. 2010 | 1007 | Thumper usage, systems exclusion^†^ |
| Taylor et al. 1978 | 50 | Publication period exclusion |
| Kim et al. 2019 | 820 | Systems exclusion |
| Liu et al. 2010 | 85 | Systems exclusion, biochemical markers were the studied outcome |
| Kurowski et al. 2014 | 150 | CardioPump feedback usage, Quality of CPR was the studied outcome, letter to the editor |
| Maule Y et al. 2007 | 290 | No English publication found |
| Ornato et al. 2005 | 1086 | Only abstract published |
| Swanson et al. 2006 | 876 | Only abstract published |
| Wilde et al. 2008 | 220 | Only abstract published |
| Paradis et al. 2009 | 1024 | Only abstract published |
| Truhlar et al. 2010 | 30 | Only abstract published |
| Morozov et al. 2012 | 188 | Only abstract published |
| Lin et al. 2015 | 455 | Thumper device usage, systems exclusion |
| Buckler et al. 2016 | 80,681 | No matching of case-control, all mechanical devices used, no demography specified or excluded, letter to the editor |
| Ananthraman et al. 2017 | 1178 | Systems exclusion |
| Hayasida et al. 2017 | 6537 | Systems exclusion |
| Hardig et al. 2017 | 757 | A subset of the LINC trial results already included in Rubertsson et al. 2014 study |

*ACD- CPR: Active Compression- Decompression device CPR

**Publication period exclusion: Published before 2000 or after 2020

^†^Systems exclusion: Study conducted outside of advanced American or European EMS systems
